# Inhibition of African Swine Fever Virus Replication by Porcine Type I and Type II Interferons

**DOI:** 10.3389/fmicb.2020.01203

**Published:** 2020-06-04

**Authors:** Wenhui Fan, Pengtao Jiao, He Zhang, Teng Chen, Xintao Zhou, Yu Qi, Lei Sun, Yingli Shang, Hongfei Zhu, Rongliang Hu, Wenjun Liu, Jing Li

**Affiliations:** ^1^CAS Key Laboratory of Pathogenic Microbiology and Immunology, Institute of Microbiology, Chinese Academy of Sciences, Beijing, China; ^2^Institute of Military Veterinary Medicine, Academy of Military Medical Science, Changchun, China; ^3^University of Chinese Academy of Sciences, Beijing, China; ^4^Shandong Provincial Key Laboratory of Animal Biotechnology and Disease Control and Prevention, College of Veterinary Medicine, Shandong Agricultural University, Tai’an, China; ^5^Institute of Animal Science, Chinese Academy of Agricultural Sciences, Beijing, China; ^6^Institute of Microbiology, Center for Biosafety Mega-Science, Chinese Academy of Sciences, Beijing, China

**Keywords:** African swine fever virus, recombinant, porcine interferon, antiviral virus activity, MHC molecules

## Abstract

Interferons (IFNs) are proteins produced by a variety of cells during the process of virus infection. It can activate the transcription of multiple functional genes in cells, regulate the synergistic effect of multiple signaling pathways, and mediate a variety of biological functions such as antiviral activity and immune regulation. The symptoms of hosts infected with African swine fever virus (ASFV) depend on the combined interaction between viruses and the host. However, it is unclear whether IFNs can be used as an emergency preventive treatment for ASFV. This study focused on the use of recombinant porcine IFNs, produced by *Escherichia coli*, to inhibit the replication of ASFV. The activity of IFN against ASFV was detected using primary alveolar macrophages at different doses through immunofluorescence assays and quantitative real-time PCR. We found that both 1000 and 100 U/mL doses significantly inhibited the replication of ASFV. Meanwhile, we found that IFNs could significantly trigger the production of a variety of IFN-induced genes (*IFIT1*, *IFITM3*, *Mx-1*, *OASL*, *ISG15*, *PKR*, *GBP1*, *Viperin*, *BST2, IRF-1*, and *CXCL10*) and MHC molecules, which play key roles in resistance to virus infection. Peripheral blood samples were also obtained from surviving pigs treated with IFNs, and the viral load was determined. Consistent with *in vitro* tests, low-dose (10^5^ U/kg) recombinant porcine IFNs (PoIFN-α and PoIFN-γ) significantly reduced viral load compared to that with high-dose (10^6^ U/kg) treatment. Our results suggest that recombinant porcine IFNs have high antiviral activity against ASFV, providing a new strategy for the prevention of African swine fever.

## Introduction

African swine fever virus (ASFV) is an enveloped, icosahedral, double-stranded DNA virus that infects the macrophages of domestic swine (Galindo and Alonso, 2017). The virus is the only member of the Asfarviridae family and has a complex molecular structure and a genome that mainly replicates in the cytoplasm of infected cells (Dixon et al., 2013). African swine fever (ASF) is a highly contagious hemorrhagic viral disease of domestic and wild pigs (*Sus scrofa*; Yutin et al., 2009). It infects animals with a ≥90% mortality rate and causes serious economic and production losses worldwide (Parker et al., 1969; Thomson et al., 1980). As of December 2019, ASF epidemics in China have resulted in the culling of over one million pigs. Outbreaks of ASFV affect pork production and consumption, as well as the annual number of pigs slaughtered (Esparza et al., 1988).

Interferons (IFNs) are key components of the response of the innate immune system to viral infection. The IFN-mediated innate immune response, selected by evolution, provides a robust first line of defense against most invading pathogens. Following pathogen detection and subsequent IFN production in the host, IFNs bind their receptors and initiate a signaling cascade, leading to the accurate transcriptional regulation of hundreds of IFN-stimulated genes (ISGs). This leads to a remarkable antiviral state in the host, which is effective against RNA and DNA viruses (Schoenborn and Wilson, 2007; Gonzalez-Navajas et al., 2012). There are reports that IFN can be used as an emergency preventive agent against outbreaks of foot and mouth disease. However, it is unclear whether IFN can be used in the same manner to prevent AFSV.

As major members of the IFN family, IFN-α and IFN-γ play important roles in innate immunity against various viral infections (Sainz and Halford, 2002; Garcia-Sastre and Biron, 2006; Liu et al., 2013). IFN-α is secreted by most cells during most viral infections (Samuel, 1998), and through binding to its heterodimeric receptor (IFNAR1 and IFNAR2), it triggers the expression of many ISGs via activation of the JAK-STAT pathway (Sadler and Williams, 2008; Schneider et al., 2014). In contrast, IFN-γ production is largely restricted to cells of the immune system (Sadler and Williams, 2008). However, IFN-γ receptors (IFNGR1/2) are widely expressed, and therefore, nearly all cell types are capable of responding to IFN-γ (Valente et al., 1992). Unlike IFN-α, after binding to its receptors, IFN-γ activates JAKs, causing the phosphorylation of STAT1 and the formation of homodimers. Then, the dimer enters the nucleus and activates GAS (gamma-activated sequence) elements to induce the expression of ISGs (O’Shea et al., 2015).

Existing studies demonstrate that human IFN-α, human IFN-γ, and bovine IFN-α inhibit ASFV replication in Vero cells or primary alveolar macrophages (PAMs; Esparza et al., 1988; Paez et al., 1990). In addition, porcine IFN-α and IFN-γ can inhibit the replication of many kinds of viruses such as classical swine fever virus, porcine reproductive and respiratory syndrome virus, Japanese encephalitis virus, and porcine epidemic diarrhea virus, *in vitro* or *in vivo* (Xia et al., 2005; Liu et al., 2013; Fernandez-Sainz et al., 2015; Fu et al., 2016; Shi et al., 2016; Brockmeier et al., 2017; Ji et al., 2017). However, the overall circumstances associated with the innate immune response, the pathways and types of IFNs that play a dominant role in innate immunity against ASFV infection, and how these processes are regulated remain unclear.

In the present study, we investigated the antiviral effect of recombinant porcine IFNs (PoIFN-α and PoIFN-γ) produced in *Escherichia coli* and their use as an emergency treatment for ASFV-positive pigs. We also used this approach to study infection in the host and variations in the production of IFNs and ISGs. These data provide new insights into the host innate immune response, and especially the multifunctional IFN regulatory mechanisms that respond to ASFV infection.

## Materials and Methods

### Ethics Statement

The pig experimental design and protocols used in this study were approved by the regulations of the Institute of Microbiology, Chinese Academy of Sciences Research Ethics Committee (Permit Number: PZIMCAS2019001). Samples were collected for ASF testing and surveillance under the agreement between the Ministry of Agriculture and Rural Affairs of the Chinese Government. Sample collection and treatment were conducted in accordance with the protocols established by the World Organization for Animal Health. The protocol was approved by the Ethics Committee of the Military Veterinary Research, Academy of Military Medical Sciences. Experiments on pigs were carried out in a BSL-3 level laboratory in the Institute of Military Veterinary Medicine, Academy of Military Medical Science. The viruses were inactivated in a BSL-3 level laboratory, and the inactivated samples were transferred to a BSL-2 level laboratory for genomic DNA extraction and detection.

### Cell Lines, Antibodies, and Virus Preparation

Primary alveolar macrophages were collected from 35-day-old pigs, used to amplify ASFV, and then grown in RPMI 1640 medium (Thermo Fisher Scientific, United States) supplemented with antibiotics (100 U/mL of penicillin, 100 mg/mL of streptomycin, and 0.25 mg/mL of Fungizone) and 10% heat inactivated fetal bovine serum (FBS, Hyclone) at 37°C with 5% CO_2_. Porcine kidney 15 cells (PK15), Madin-Darby bovine kidney cells (MDBK), and Madin-Darby Canine Kidney cells (MDCK) were maintained in our laboratory and grown in DMEM medium (Thermo Fisher Scientific, United States) supplemented with antibiotics (100 U/mL of penicillin and 100 mg/mL of streptomycin) and 10% heat inactivated FBS at 37°C with 5% CO_2_. Goat anti-rabbit IgG monoclonal antibody and rabbit anti-P30 polyclonal antibody were purchased from Alpha Diagnoestic International (ASFV11-C). The virus, ASFV strain SY18 of genotype II (GenBank accession number MH766894), was stored at the Institute of Military Veterinary Medicine, Academy of Military Medical Science. The viral titer was determined based on macrophage cultures (TCID_50_/mL). The ASFV P72 proteins were detected by commercial ELISA kit (YaJibiological, China. Cat. No: YS07258B).

### Expression and Purification of Recombinant PoIFN-α and PoIFN-γ

Protein expression and purification were performed as previously described with minor modifications (Meng et al., 2011). *E. coli.* strain Rosetta (DE3) was transformed with the recombinant expression plasmid pET30a-His-PoIFN-α or pET28a-His-PoIFN-γ (Liu et al., 2019), and a single colony was cultured in LB medium at 37°C until the OD_600_ reached 0.5. Protein production was then induced with 1 mM IPTG for 8 h at 37°C. The cells were collected by centrifugation and precipitation and resuspended in PBS for sonication. Then, inclusion bodies were isolated by centrifugation at 4°C and 10,000 × *g* for 10 min, and recombinant PoIFNα and PoIFN-γ were purified by gel filtration. After purification, the denatured proteins were refolded. Purity was assessed by SDS-PAGE and western blotting, and the concentrations of the recombinant protein were determined using a BCA protein assay kit (CW Bio) according to the manufacturer’s instructions. The recombinant PoIFN-α and PoIFN-γ preparations were treated to remove lipopolysaccharide using a ToxinEraser Endotoxin Removal Kit (GenScript), following the manufacturer’s directions. The proteins were then diluted and filtered through a 0.22-μm membrane and stored at 4°C.

### Detection of Toxicity of Interferon at Different Doses

PAM cells seeded in 96-well cell culture plates were treated with IFNs at final concentrations ranging from 1 to 1000 U/mL, then the treated cells were incubated for 72 h at 37°C under 5% CO_2_ condition. After incubation, the medium was removed and PAM cells were stained with 0.4% crystal violet ethanol solution for 30 min followed by washing with distilled water. Colorimetric measurement was done by a microplate reader at 590 nm wavelength. The percentage of viable cells was evaluated by each concentration as [(ODTreated/ODNC) × 100], where ODTreated and ODNC were the absorbance of treated and control PAM cells, respectively.

### Determination of the Antiviral Activity of PoIFN-α and PoIFN-γ *in vitro*

The biological antiviral activity of *E. coli*-derived PoIFN-α and PoIFN-γ was evaluated via the cytopathic effect (CPE) inhibition method based on VSV/PK15, VSV/MDBK, and VSV/MDCK systems according to previously described protocols (Armstrong, 1971; Taira et al., 2005). Cells were grown in 96-well plates until they reached monolayer status at 37°C with 5% CO_2_. Then, the cells were washed with warm sterile PBS and stimulated with 100 μL of four-fold serially-diluted PoIFN-α and PoIFN-γ for 12 h; the cells were challenged with 100 TCID_50_ viruses per well and cultured until a CPE was observed in virus-infected cells without PoIFN-α. Cells were finally stained with crystal violet, and the OD_570_ was measured using a Microplate Reader (Thermo Fisher Scientific). The PoIFN-α/PoIFN-γ titers (U/mg) were expressed based on the reciprocal of the dilutions that led to 50% virus-induced cell lysis via the *Reed-Muench* method.

For ELISA experiment, the cell culture supernatant was collected at 72 h after infection. The ASFV P72 proteins were detected by commercial ELISA kit according to the manufacturer’s protocol and the optical density (OD) value was assayed by a colorimetric reader. The OD value was measured at 450 nm wavelength. Samples with an OD value higher than 0.2 were considered positive for ASFV.

### Indirect Immunofluorescence Assay

Primary alveolar macrophages were seeded in 24-well plates with slides at a concentration of 2 × 10^6^/mL and incubated for 12 h at 37°C with 5% CO_2_ in RPMI 1640 maintenance medium. Then, the cells were stimulated with PoIFN-α and PoIFN-γ for 12 h before ASFV infection. The cells were infected with SY18 (MOI = 1) and were analyzed by immunofluorescence assay (IFA) at 48 h post-infection. In brief, the cells were fixed with 4% paraformaldehyde at 4°C overnight, and the fixed cells were permeabilized with 0.5% triton X-100 in PBS (PBST) for 20 min at 25°C and blocking buffer (PBST with 4% BSA) for 1 h at 37°C. The cells were incubated with rabbit anti-P30 polyclonal antibody (100-fold diluted in blocking buffer) for 2 h at 37°C, followed by three washes with PBST and incubation with FITC-conjugated goat anti-rabbit IgG monoclonal antibody (100-fold diluted in blocking buffer) for 1 h at 37°C. DAPI (1000-fold diluted) was used to stain the nuclei for 20 min at 25°C, and the cells were observed using a confocal laser scanning fluorescence microscope (Olympus LSCMFV500).

### Flow Cytometric Analysis of p30 Indirect Immunofluorescence

Primary alveolar macrophages were seeded in 6-well plates at a concentration of 2 × 10^6^/mL and incubated for 12 h at 37°C with 5% CO_2_ in RPMI 1640 maintenance medium. Then, the cells were stimulated with PoIFN-α and PoIFN-γ for 12 h when ASFV infection. The cells were infected with ASFV strain SY18 (MOI = 1) and were digested and harvested by centrifugation at 48 h post-infection. Then, the cells were fixed with 5 mL 4% paraformaldehyde at 4°C overnight, and the fixed cells were centrifuged and permeabilized with 5 mL 0.5% PBST for 20 min at 25°C and blocking buffer (PBST with 4% BSA) for 1 h at 37°C. Next step, the cells were incubated with rabbit anti-P30 polyclonal antibody (100-fold diluted in blocking buffer) for 2 h at 37°C, followed by three washes with PBST and incubation with FITC-conjugated goat anti-rabbit IgG monoclonal antibody (100-fold diluted in blocking buffer) for 1 h at 37°C. After three washes with PBST the cells were analyzed by analyzed using a FACS Calibur flow cytometer (BD Biosciences). Data were analyzed with Flowjo software (Treestar Inc).

### *In vitro* Infection

Primary alveolar macrophages were seeded in 96-well plates at a concentration of 2 × 10^6^/mL and incubated for 12 h at 37°C with 5% CO_2_ in RPMI 1640 maintenance medium. The infected PAM samples were diluted with RPMI 1640 medium (without FBS) to generate serial 10-fold dilutions to 10^–8^. RPMI 1640 maintenance medium was removed and then 100 μL of each sample dilution was added (six wells per dilution) to the wells, which were then incubated at 37°C for 1 h. After incubation, the virus suspensions were removed and 100 μL of RPMI 1640 maintenance medium was added to each well. The infected PAM cells were collected and stored at −80°C.

### TaqMan PCR Assay

African swine fever virus genomic DNA was extracted using GenElute^TM^ Mammalian Genomic DNA Miniprep Kits (Sigma Aldrich, United States) from PAMs with different treatments or EDTA-treated whole pig peripheral blood. TaqMan PCR assays were performed on an Applied Biosystems 7500 Real Time Detection System (Roche, Germany) according to the OIE-recommended procedure described by King et al. (2003). DNA templates from different treatment groups were adjusted to the same concentrations in both cell and animal experiments before the TaqMan PCR assay to increase the reliability of P72ct values. The ΔCT in the animal experiment was calculated by subtracting the lower CT value from the higher CT value of the same animal of different time point.

### Quantitative Real-Time PCR (q-PCR) Assay

Primary alveolar macrophages were grown in 12-well plates at a concentration of 2 × 10^6^/mL and incubated for 12 h at 37°C with 5% CO_2_ in RPMI 1640 maintenance medium; then Primary alveolar macrophages were incubated with the indicated dose of PoIFN-α and PoIFN-γ. After the incubation, PAMs were harvested using TRIzol Reagent according to the manufacturer’s instructions (Invitrogen). Total RNA was extracted, and its quality and quantity were determined using a NanoDrop 1000 spectrophotometer (Thermo-Fisher Scientific). Then, the RNA was reverse-transcribed to cDNA using the Reverse Transcription System (Promega). It performed in accordance with the manufacturer’s instructions.

The cDNA was quantified with a TB Green Advantage qPCR Premix (Takara) on an Applied Biosystems 7500 Real Time Detection System (Roche, Germany) to determine the gene expression levels under the control of the β*-actin* gene promoter. The primers are shown in Supplementary Table S1. cDNA from PBS-treated PAMs was used as a calibrator to evaluate the mRNA levels of genes encoding ISGs. Each assay was performed in triplicate and the mRNA level was calculated by 2^–ΔΔct^ method. ASFV genomic DNA was extracted using GenElute Mammalian Genomic DNA Miniprep Kits (Sigma Aldrich, United States) from PAMs with different treatments or EDTA-treated whole pig peripheral blood.

### Administration of IFNs and Pig Test

Eleven 40-day-old pigs weighing ∼15 kg were screened from free-range households. All pigs tested negative for anti-ASFV antibodies, as determined by a commercial ELISA kit (ASFV Ab Test, ID.VET). The pigs also tested negative for classical swine fever virus, respiratory syndrome virus, porcine circovirus type 2, porcine reproductive and pseudorabies virus, and influenza A virus by PCR or RT-PCR. Specific primers for the detection of viruses are described in previous studies (Ogawa et al., 2009; Hu et al., 2015). Pigs were randomly separated into three groups (LDI, HDI, and Untreated), with 3–4 pigs per group, and housed in a BSL-3 laboratory.

The challenged pigs were orally inoculated with 10^2^ TCID_50_ SY18. Rectal temperatures and clinical signs were recorded daily during the experiment for each group. The pigs were injected with IFNs via intramuscular injection at intervals of 24 h after challenge, for a total of three times (diluted to 1 mL/pig). EDTA-stabilized blood samples were collected for the extraction of total DNA, which were then used to determine P72 gene expression profiles at 1, 10, 20, and 30 days post-immunization based on the TaqMan PCR assay described previously herein.

### Statistical Analyses

Statistical comparisons were performed using GraphPad Prism version 6.0 (GraphPad software Inc.). Comparisons between groups were made with a Student’s *t* test. Data are expressed as the mean ± standard deviation (SD). Differences with *P* < 0.05 were considered statistically significant.

## Results

### PoIFN-α and PoIFN-γ Exert Different Antiviral Activities

A prokaryotic expression system was used to produce recombinant PoIFN-α and PoIFN-γ, which were purified by Ni-affinity chromatography. The antiviral activities of PoIFN-α and PoIFN-γ were determined according to the CPE inhibition method based on the VSV/PK15, VSV/MDBK, and VSV/MDCK systems and according to previously described protocols (Armstrong, 1971; Taira et al., 2005). PoIFN-α and PoIFN-γ demonstrated unequal biological activities in different virus/cell systems, such as higher antiviral activity in the VSV/MDBK and VSV/PK15 systems than in the others. PK15 is pig kidney cell line, and the result of PK15 cell lines shows its species specific antiviral activity, while IFN has a broad spectrum of antiviral properties, there is also antiviral activity effect in MDBK and MDCK. Our analysis suggests that this may be related to the cross-species antiviral activity of pig IFN. These suggestions have also been reported in the previous literature (Ghaffar et al., 1992; Kubes et al., 1994).The antiviral activity of PoIFN-α was better than that of PoIFN-γ in all systems (Table 1). The endotoxin levels of PoIFN-α and PoIFN-γ proteins showed that the contents of endotoxin were all lower than 0.0003 EU/μg, which effectively avoided the induction of cellular immune response in cell experiments due to the presence of endotoxin, making the experimental results more reliable (Supplementary Table S2). The protein purity was analyzed by SDS-PAGE and the results were shown in Supplementary Figure S1.

**TABLE 1:** Antiviral activities of IFN-α and IFN-γ in different cells.

**Interferon**	**Antiviral activity (× 10^6^U/mg) in the cells**		
	**VSV/PK15**	**VSV/MDBK**	**VSV/MDCK**
IFN-α	12.3	210.3	0.056
IFN-γ	5.5	25.6	0.0025

*Data are shown as the mean of three independent experiments. Data are means (*n* = 3) ± SEM.*

### PoIFN-α and PoIFN-γ Inhibit the Replication of ASFV in PAMs With a Synergistic Effect

**FIGURE 1:**
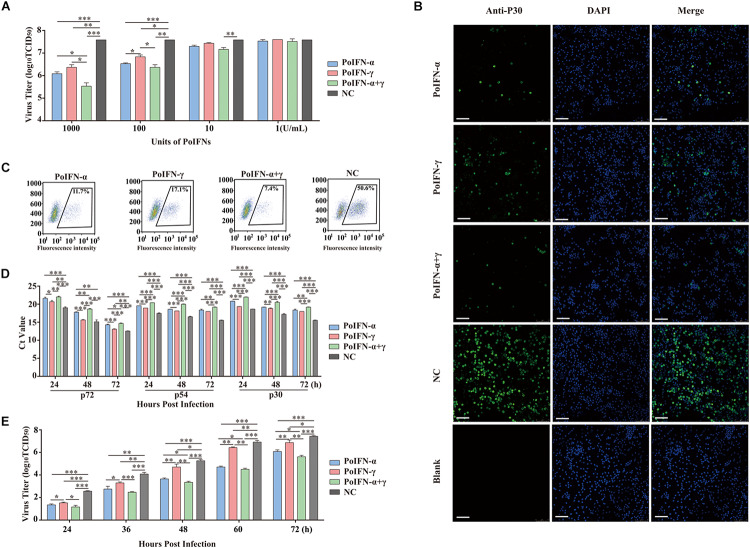
PoIFN-α and PoIFN-γ inhibit the replication of African swine fever virus (ASFV) in primary alveolar macrophages (PAMs). **(A)** IFN inhibited virus replication. Primary alveolar macrophages were stimulated with PoIFN-α and PoIFN-γ at the indicated concentrations for 12 h in 24-well plates, and then the cells were infected with ASFV strain SY18 for 1 h at an MOI of 1. After removing the virus, PAMs were further cultured for 72 h prior to sample collection. **(B)** Indirect immunofluorescence analysis of anti-P30 expression in PAMs. The cells were exposed to a concentration of 1000 U/mL PoIFN-α, PoIFN-γ, or those combined for 12 h prior to infection with ASFV strain 18 for 1 h at an MOI of 1, maintained for 72 h after adsorption without PoIFN-α or PoIFN-γ, washed, and fixed with 4% paraformaldehyde. P30 expression was detected with a rabbit polyclonal antibody and FITC-conjugated AffiniPure Goat Anti-Rabbit IgG (H + L), and the nucleus was stained with DAPI. Scale bars represent 75 μm. **(C)** The antivial of IFNs against ASFV by immunoblot analysis. The PAM cells were treated with IFNs and then were infected with SY18 and collected for indirect immunofluorescence 48 h after infection. Next step, flow cytometric were used to analyze the proportion of fluorescent cells. 10^4^ cells were selected and the number of fluorescent cells was counted for the purpose of counting the proportion of fluorescent cells. **(D)** The effect of PoIFN-α and PoIFN-γ on ASFV replication in PAMs detected by TaqMan qPCR methods. Primary alveolar macrophages were exposed to a fixed optimal concentration of 1000 U/mL PoIFN-α, PoIFN-γ, or those combined for 12 h prior to infection with SY18 for 1 h at an MOI of 1 and maintained for different times after adsorption without PoIFN-α or PoIFN-γ; the ASFV P72 Ct value of different groups at the indicated time points was determined. **(E)** Growth curve of SY18 in PAMs. Primary alveolar macrophages were exposed to a fixed optimal concentration of 1000 U/mL PoIFN-α, PoIFN-γ, or those combined for 12 h prior to infection with SY18 for 1 h at an MOI of 1 and maintained for different times after adsorption without PoIFN-α or PoIFN-γ, and the virus titer was determined in PAMs by TCID_50_ assays. Three replicates were performed for all analyses. Bars represent the means ± SD (*n* = 3). The data of 0 h detection point is not shown. A non-parametric test was used for the difference significance analysis. Statistically significant differences are indicated (^∗^*P* < 0.05; ^∗∗^*P* < 0.01; ^∗∗∗^*P* < 0.001) and the line above the column marks the two groups with differences.

Generally, ASFV infects immune cells of the myeloid lineage and mainly grows and is amplified *in vitro* in PAMs and peripheral blood mononuclear cells (Sanchez-Cordon et al., 2008). To determine the replication efficiency of ASFV treated *in vitro* with different doses of PoIFN-α and PoIFN-γ, PAMs were infected with SY18 (MOI = 1). The antiviral activity of various doses of PoIFN-α and PoIFN-γ against SY18 infection in PAMs was evaluated. The viral titers of 1000 U/mL doses of PoIFN-α, PoIFN-γ and combination treatment group were 10^6.08^, 10^6.37^ and 10^5.53^ TCID_50_/mL respectively, while the viral titer of negative control (NC) group was 10^7.58^. The differences between the IFN treatment groups and NC group were extremely significant (*P* < 0.001). The viral titers of 100 U/mL doses of PoIFN-α, PoIFN-γ and combination treatment group were 10^6.53^, 10^6.83^, and 10^6.36^ TCID_50_/mL respectively (Figure 1A). The expression of P30 was determined by IFA, and the intensity of fluorescence and the proportion of fluorescent cells reflected the inhibitory effect of different IFN treatments on the virus (Figure 1B). Flow cytometry was used to determine the proportion of fluorescent cells in immunofluorescence experiments. The proportion of fluorescent cells of PoIFN-α, PoIFN-γ, combination treatment group and NC group were 11.7, 17.1, 7.4, and 50.6% respectively (Figure 1C) and the differences between treatment groups and NC group were significant (*P* < 0.001). The anti-ASFV activity of porcine IFNs was evaluated by comparing the proportion of green fluorescent cells between the IFN treated and negative control group, consistent with Figure 1A. Aliquots of cell supernatants were harvested at 24, 48, and 72 h post-infection. As an important structural protein produced in late stage of viral infection, P72 is crucial for the antigenicity and formation of virus capsid. P30 is an important membrane protein which produced in 2–4 h after infection and continuously expressed throughout the whole infection period. P54 is also membrane protein produced in the early stage of infection and plays an important role in virus adsorption to susceptible cells and invasion (Salas and Andrés, 2013; Lithgow et al., 2014; Sánchez et al., 2017). P30, P54, and P72 are rich structural proteins in ASFV, and their expression level can represent the amount of virus to some extent. The inhibition of SY18 by the 1000 U/mL dose was further confirmed by measuring the Ct value of the P72, P30 and P54 genes using TaqMan PCR assays and by evaluating the TCID_50_ of the virus at different infection times by IFA. The change trend of P30 and P54 genes was detected, which was the same as that of P72, and further supported the antiviral activity of pig IFNs against ASFV (Figure 1D). For ELISA experiment, IFN levels in PAM culture supernatants collected from IFN treated group or NC group, the result showed that IFN-α and IFN-γ combination is higher Antiviral activity in the cells, it is consistent with the trend of CT value (Supplementary Tables S3,S4). Basically the same as the results shown in Figure 1A, we found that a 1000 U/mL dose of PoIFN-α and PoIFN-γ, alone or combined, resulted in significant inhibition, and combined treatment resulted in the most significant inhibition (Figure 1E).

Replication analysis revealed that the propagation of SY18 was robust in PAMs over a 24-h period, reaching titers of approximately 2.5 log_10_ TCID_50_/mL. These results were compared to those of the IFN-treated group, where the virus replication slowed significantly. Combined PoIFN-α and PoIFN-γ treatment resulted in the most significant differences. The results showed that there was no difference in the percentage of viable cells in each group, indicating that the current dose of IFN would not produce cytotoxicity. In addition, IFN will be short half-life by the body will be quickly metabolized, will not cause toxic effects (Supplementary Figure S2).

### PoIFN-α and PoIFN-γ Induces the Expression of ISGs in PAMs

**FIGURE 2:**
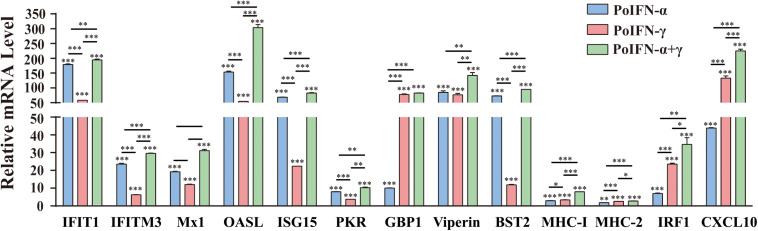
Gene expression in primary alveolar macrophages (PAMs) induced with PoIFN-α and PoIFN-γ. Fresh PAMs were isolated from healthy 35-day-old pigs and cultured in RPMI 1640 medium with 10% FBS and 5% CO_2_ for 12 h. Then, the cells were treated with PoIFN-α or PoIFN-γ for 12 h. After the 12 h, the cells were harvested and lysed in TRIzol Reagent for RNA extraction. The vertical axis shows the relative transcription level (fold-change) of IFN-stimulated gene (ISG) expression comparing the negative control and treated groups. The fold-changes were measured by the 2^–ΔΔCt^ method, and RNA levels were normalized. Three replicates were performed for q-PCR verification analyses. Bars represent the means ± SDs (*n* = 3). A non-parametric test was used for the difference significance analysis. Statistically significant differences are indicated (^∗^*P* < 0.05; ^∗∗∗^*P* < 0.01; ^∗∗∗^*P* < 0.001) and the line above the column marks the two groups with differences.

To explore the effect of PoIFN-α and PoIFN-γ on the expression of ISGs, the optimal concentration of 1000 U/mL of PoIFN-α and PoIFN-γ was applied to PAMs, and quantitative real-time PCR (q-PCR) was employed to measure the mRNA expression of *IFIT1*, *IFITM3*, *Mx1*, *OASL*, *ISG15*, *PKR*, *GBP1*, *Viperin*, *BST2*, *IRF1*, and *CXCL10* after a 12-h incubation (Figure 2). We found that PoIFN-α and PoIFN-γ significantly induced the expression of multiple ISGs in PAMs. Compared to PoIFN-γ, PoIFN-α was found induce a higher level of ISG transcription overall. However, PoIFN-γ had a more significant induction effect on some individual ISGs such as *GBP* and IFN-γ stimulated genes such as *IRF1*, particularly. Additionally, ISGs induced by the combination of PoIFN-α and PoIFN-γ was most significant compared to that with each separately.

To identify the role of PoIFN-α and PoIFN-γ in improving cell immunity, we also monitored the transcription levels of MHC-I and MHC-II molecules in PAMs treated with IFN. We found that the transcriptional levels of MHC-I and MHC-II did not increase, as shown for the other ISGs, but that treatment with PoIFN-α and PoIFN-γ combined could significantly induce MHC-I transcription.

### Combined PoIFN-α and PoIFN-γ Decreases the ASFV Viral Load in Challenged Pigs

**FIGURE 3:**
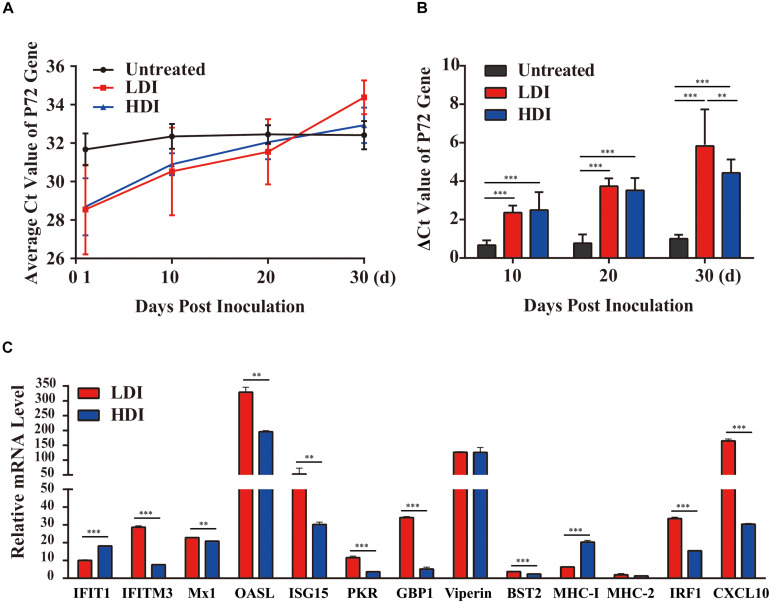
African swine fever virus (ASFV) P72 Ct values at the indicated time points in PoIFN-α- and PoIFN-γ-treated surviving pigs after ASFV infection. Groups included PBS (Untreated), low-dose PoIFN-α and PoIFN-γ combined (LDI, 1 × 10^6^ U/kg), and high-dose PoIFN-α and PoIFN-γ combined (HDI, 1 × 10^7^ U/kg). **(A)** The average Ct value of the P72 gene was recorded for each group. **(B)** The increase in the ΔCt value of the P72 gene was determined for each group. Data are representative of the mean ± SDs (*n* = 3). A non-parametric test was used for the difference significance analysis. **(C)** Gene expression in pig peripheral blood lymphocyte induced with PoIFN-α and PoIFN-γ. Anticoagulant peripheral blood was drawn from pig and lysed in TRIzol Reagent for RNA extraction. The vertical axis shows the relative transcription level (fold-change) of IFN-stimulated gene (ISG) expression comparing the negative control and treated groups. The fold-changes were measured by the 2^−ΔΔ*Ct*^ method, and RNA levels were normalized. Three replicates were performed for q-PCR verification analyses. Bars represent the means ± SDs (*n* = 3). A non-parametric test was used for the difference significance analysis. Statistically significant differences are indicated (^∗∗^*P* < 0.01; ^∗∗∗^*P* < 0.001) and the line above the column marks the two groups with differences.

To verify the antiviral effect of combined PoIFN-α and PoIFN-γ in pigs, different doses of IFN were administered to ASFV challenged pigs. Using TaqMan PCR methods followed by the ARRIVE guidelines (Kilkenny et al., 2010), the blood of all pigs was tested for ASFV P72 transcripts before and after treatment with combined PoIFN-α and PoIFN-γ at different time points (Figure 3A). We found that combined PoIFN-α and PoIFN-γ treatment significantly reduced the Ct value of the P72 gene in the challenged pigs compared to that in the untreated group (Table 2). The data was shown that both high-dose IFN treatment and low-dose IFN treatment reduced the amount of virus in pigs, and the effect in the low-dose group was better than that in the high-dose group (Figure 3B). Some of ISGs expressions after IFN treatments in pigs by qPCR as shown in Figure 3C. After the stimulation of PoIFN, the ISGs expression was up-regulated, obviously, the increase was sharper in the low-dose group, and the difference was significant or extremely significant between low-dose group, except for *Viperin* and *MHC-2* (Figure 3C). After inoculation, the IFN levels in serums were measured by cytopathic inhibition method based on PK15/VSV system. According to the test data, serum IFN levels decreased significantly over time, and the results showed that the IFN-α and IFN-γ combination group had a better effect, with an activity of 121.21 U/mL at 4 h, shown as Supplementary Table S5.

**TABLE 2:** CT values of P72 in pigs treated with low-dose and high-dose interferons.

**No.**	**Day 1**			**Day 10**			**Day 19**			**Day 28**		
Untreated-1	31.55	31.79	31.67	32.38	32.49	32.44	32.53	32.76	32.65	32.70	32.75	32.73
Untreated-2	32.74	32.62	32.68	33.15	32.87	33.01	33.15	33.21	32.68	32.96	33.74	33.35
Untreated-3	30.32	30.65	30.48	31.06	31.70	31.38	31.94	31.83	31.88	31.59	31.75	31.67
Untreated-4	32.06	31.66	31.86	32.44	32.69	32.56	31.97	32.62	32.29	32.20	31.58	31.89
LDI-1	26.20	25.98	26.09	28.91	28.73	28.82	30.03	29.89	29.96	33.77	33.87	33.82
LDI-2	28.72	28.80	28.76	30.56	30.53	30.55	31.71	31.73	31.72	33.69	33.91	32.90
LDI-3	31.95	32.13	32.04	34.82	33.26	34.04	34.10	34.14	34.12	35.15	35.93	35.54
LDI-4	27.26	27.30	27.28	28.71	28.68	28.70	30.20	30.58	30.39	34.42	34.90	34.66
HDI-1	31.03	31.25	31.14	31.57	31.35	31.46	33.10	31.93	32.51	33.38	33.92	32.65
HDI-2	27.77	27.59	27.68	31.65	30.60	31.12	31.88	31.94	31.91	31.69	31.63	31.66
HDI-3	27.99	28.14	28.07	29.97	30.27	30.12	30.76	30.84	30.80	32.91	32.65	32.78
HDI-4	27.86	27.89	27.87	31.00	30.80	30.90	32.97	32.93	32.95	33.95	33.95	33.95

*HDI, high-dose interferon (IFN) treatment; LDI, low-dose IFN treatment.*

## Discussion

As with other viruses, the signs of hosts infected with ASFV depend on the combined interaction between viruses and the host. The natural ASFV hosts, warthogs and bush pigs, usually present with persistent infections without obvious clinical signs, which might be the result of long-term adaptation (Galindo and Alonso, 2017). Acute hemorrhagic fever and high mortality are the main characteristics of ASFV-infected domestic pigs. African swine fever virus suppresses innate immunity through a variety of mechanisms, and viral infections compete with the natural immunity of the host (Reis et al., 2017). With an increasing understanding of the interaction between ASFV and hosts at the molecular, cellular, and animal levels, new insights have been provided, which will promote the further development of ASFV preventative and treatment methods, including the development of vaccines or blockers.

Interferons are one of the most important molecules of innate immunity, and these respond to viral infection. The induction of type I IFN requires pattern recognition receptors on the plasma membrane, in the cytoplasm, or on the endosome membrane to recognize pathogen-associated molecular patterns including viral DNA or RNA (Schneider et al., 2014). Viral DNA can be recognized by cGAS to catalyze the conversion of ATP and GTP to synthesize cGAMP, which then binds STING localized at the endoplasmic reticulum. In theory, ASFV is a DNA virus that replicates mainly in the cytoplasm. The DNA receptor signaling pathway plays a key role in ASFV recognition and type I IFN induction. However, viral DNA can also be transcribed into dsRNA by host RNA polymerase III or viral RNA polymerase. At present, the recognition mechanism associated with ASFV infection is still unclear.

IFNs play an antiviral role in different cell lines through the induction of different ISGs. Studies have shown that when PK15 cells are treated with PoIFN-α, the expression levels of Mx, PKR, and 2′,5′-OAS can be significantly upregulated, thus inhibiting infection by Japanese encephalitis virus (Liu et al., 2013). ASFV-encoded multigene families (MGFs) inhibit the type I IFN response (Correia et al., 2013), and the type I IFN pathway is inhibited in macrophages infected with highly pathogenic ASFV (Golding et al., 2016). According to a previous report, the expression of type I IFN and ISGs in macrophages infected with MGF360-deleted viruses is upregulated. MGF360-15R (A276R) inhibits IRF3 by inhibiting TLR3 and the cytoplasmic receptor signaling pathways but does not rely on the IRF7 and NF-κB pathways to inhibit the production of type I IFN (de Oliveira et al., 2011).

Previous studies have demonstrated that the type I IFN system plays an important role in controlling ASFV replication and inducing protective immune responses in infected pigs (Esparza et al., 1988; Paez et al., 1990). The upregulation of type I IFN expression in macrophages infected with ASFV can cause signal amplification in adjacent cells and induce ISGs to activate the innate immune response and reduce viral replication (Lacasta et al., 2015). Inducing antiviral status alone has limited effects on inhibiting the replication of ASFV. Accordingly, the activation and recruitment of natural immune cells are needed to control the replication of ASFV, which also provides time to activate and guide the adaptive immune response to eliminate viruses. Other reports have demonstrated that ASFV can inhibit the expression of type I and II IFN (Correia et al., 2013). However, *in vivo* experiments previously revealed that IFN-α/IFN-β can be detected in the serum of animals infected with highly virulent viruses (Karalyan et al., 2012).

ISGs adopt a wide range of biological activities. Many ISGs control viral, bacterial, and parasite infection by directly targeting pathways and functions required during diverse pathogen life cycles. A key component of the protective antiviral host defense is driven by the complicated intracellular actions of proteins regulated by ISGs (Fensterl and Sen, 2015). Secreted type I IFN (α/β) binds to the cell surface receptors of virus-infected cells and adjacent uninfected cells to initiate the JAK-STAT pathway and activate STAT1 and STAT2 through phosphorylation. After dimerization and binding to IRF9, the ISGF3 complex enters the nucleus to initiate the expression of ISGs (Sadler and Williams, 2008; Schneider et al., 2014). It’s reported that TRIM29 promotes DNA virus infection by inhibiting innate immune response (Xing et al., 2017). More interestingly in our test, the mRNA levels of TRIM29 in PAM cells before and after IFN treatment were no significant difference in the mRNA levels of TRIM29. The results suggested that IFNs treatment could not reduce the expression of TRIM29 to control ASFV infection.

There is discrepancy in the application of IFNs in experimental verification. Existing data shows that mammalian expressed porcine IFN α does not block replication of a number of ASFV isolates, such as Georgia 2007/1. Analysis of the discrepancy, in addition to the virus strains, there is also discrepancy in the development process of IFN. The experimental process in our study has been improved to obtain the antiviral activity of IFN. In our experiments, the PoIFN-α and PoIFN-γ expressed in *E. coli*. System with high antiviral activity was produced and could functionally inhibit the replication of SY18 in PAM cells in a way that persists in the medium.

In our study, PoIFN-α and PoIFN-γ were combined as an emergency treatment for ASFV-positive pigs and administered using a porcine model to inhibit the replication of ASFV. Primary alveolar macrophages treated with PoIFN-α and PoIFN-γ combined were collected to isolate peripheral lymphocytes, and then ISGs, MHC molecules, and virus output were assessed. Interferon-induced gene expression and immune responses were quantified and compared to those after the inoculation of ASFV without PoIFN. The results suggested that PoIFN-α and PoIFN-γ play a more effective anti-viral role. Porcine IFN functions through an IFN regulator, whereas PoIFN is more involved in activating the immune system. IFN-stimulated genes have different mechanisms to inhibit viral replication, such as binding and regulating the functions of cellular and viral proteins and RNA or DNA. Our results suggest that a combination of recombinant porcine IFN has high antiviral activity against ASFV in PAMs. Moreover, it is obvious that low-dose combined recombinant porcine IFN (10^5^ U/kg) can significantly upregulate cytokines, significantly reduce virus output, inhibit ASFV proliferation, and alleviate the clinical signs of early infection.

Although researchers have made great progress in studying the interaction between ASFV and hosts, there are still many unknowns that require further exploration. The proteomic map of ASFV provides a foundation for the study of virus invasion mechanisms, vaccine targets, and strategies to prevent and control ASFV infection. It is also a good point to method the antiviral effect of IFN from the perspective of receptor, which can be verified by subsequent experiments. MGF360 genes are important pathogenic factors that can inhibit the production and response of type I IFN (Golding et al., 2016). However, little research has been performed on the mechanisms and the function of individual genes in this family. Data are limited on the duration and pattern of the dynamics of shedding and excretion for this currently circulating ASFV strain. Guinat et al. (2014) provided quantitative data on shedding and excretion of the Georgia 2007/1 ASFV strain among domestic pigs and suggest a limited potential of this isolate to cause persistent infection, which is focus of our follow-up research. The use of porcine IFN as an antiviral agent for emergency prevention of ASF is also worthy of further study, which is the focus of our subsequent verification work in more clinical trials.

## Data AVailability Statement

Publicly available datasets were analyzed in this study. This data can be found here: GenBank accession number MH766894.

## Ethics Statement

The animal study was reviewed and approved by the Military Veterinary Research, Academy of Military Medical Sciences.

## Author Contributions

JL and WL conceived and designed the experiments. WF, PJ, and HeZ performed the IFN purification, determination of IFN activity, and immunology analyses. TC, XZ, and YQ performed the viral replication tests and animal experiments. WF, PJ, and JL performed data analyses. JL and WF prepared the manuscript and completed its revision. YS, LS, RH, and HoZ suggested many of the experiments in this study. All authors read and approved the final manuscript.

## Conflict of Interest

The authors declare that the research was conducted in the absence of any commercial or financial relationships that could be construed as a potential conflict of interest.

## Supplementary Material

FIGURE S1 The SDS-PAGE of purified PoIFN-α and PoIFN-γ.

FIGURE S2 The toxicity experiment of interferon on PAM cells.

TABLE S1 Primer sequences used in this study.

TABLE S2 Determination of Endotoxin Contamination.

TABLE S3 ELISA for ASFV P72 antigen in the cell culture supernatants collected from IFN treated group or NC group.

TABLE S4 IFN levels in PAM culture supernatants collected from IFN treated group or NC group.

TABLE S5 The IFN levels in serum after the inoculation.
