# Supplementary figures and images for: Inhibition of African Swine Fever Virus Replication by Porcine Type I and Type II Interferons
Source: Front Microbiol. 2020 Jun 4;11:1203. doi: 10.3389/fmicb.2020.01203 (PMC7325991; doi:10.3389/fmicb.2020.01203)

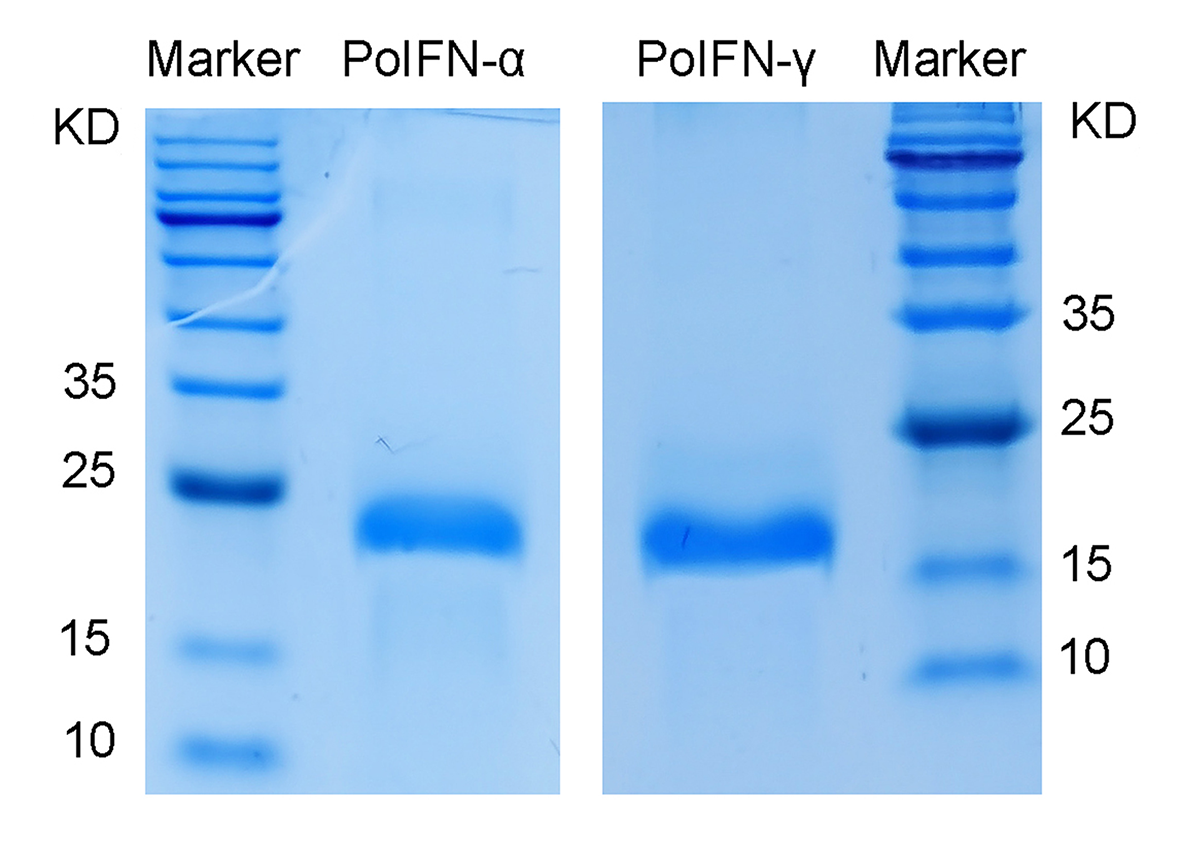

Supplement: FIGURE S1 — The SDS-PAGE of purified PoIFN-α and PoIFN-γ. [file Image_1.TIF]

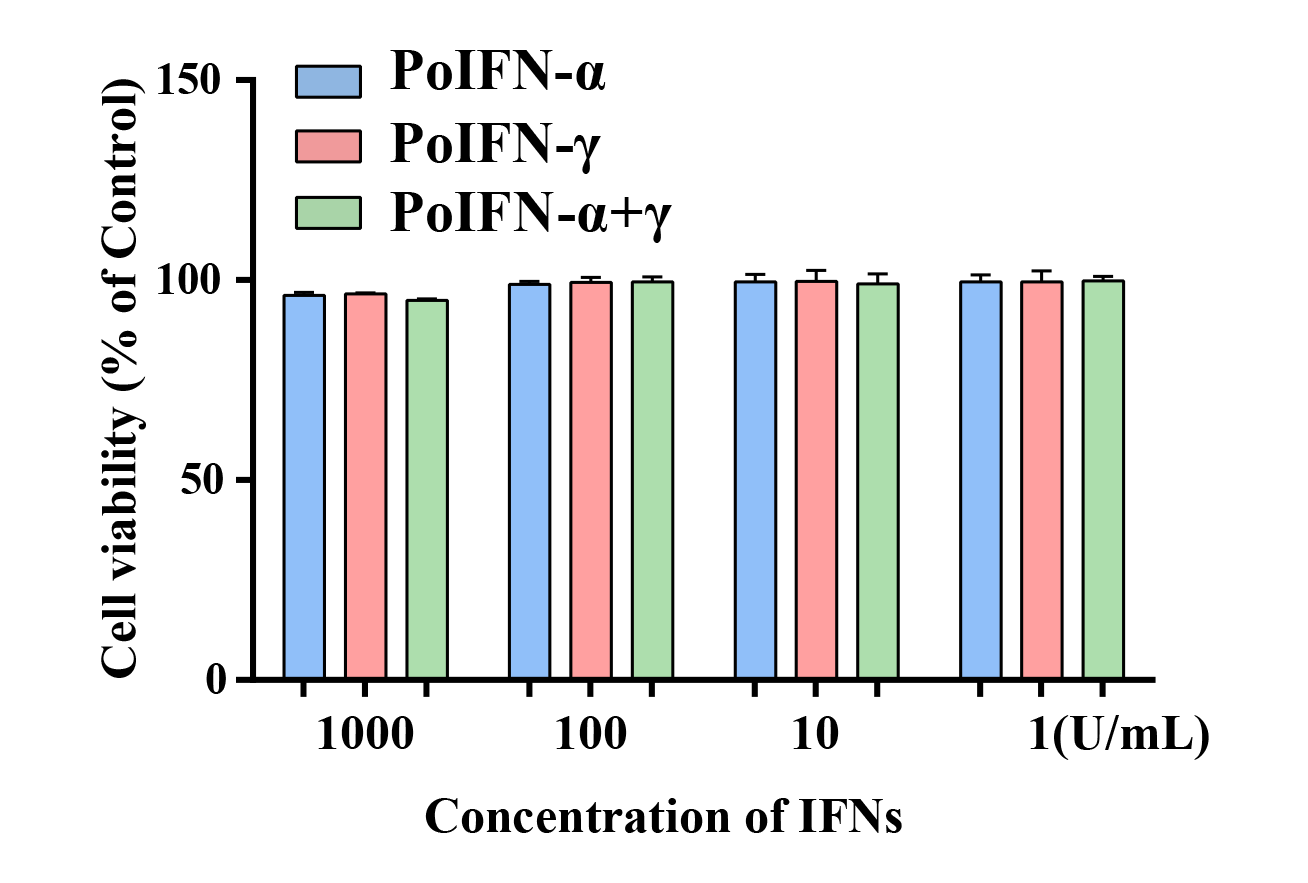

Supplement: FIGURE S2 — The toxicity experiment of interferon on PAM cells. [file Image_2.TIF]
