# Supplementary material for: Inhibition of African Swine Fever Virus Replication by Porcine Type I and Type II Interferons
Source: Front Microbiol. 2020 Jun 4;11:1203. doi: 10.3389/fmicb.2020.01203 (PMC7325991; doi:10.3389/fmicb.2020.01203)
Supplement: TABLE S1 — Primer sequences used in this study. [file Data_Sheet_1.doc]

**Supplementary Table 1 Primer sequences used in this study**

| Primer name | Primer sequences (5’-3’) |
| --- | --- |
| Pig-IFIT1-F | CTGACTCACAGCAACCATG |
| Pig-IFIT1-R | CTTTCAGGTGTTTCACATAGG |
| Pig-IFITM3 -F | GTCGTCTGGTCCCTGTTCAAC |
| Pig-IFITM3 -R | GAGTAGGCGAAAGCCACGAA |
| Pig-Mx1-F | TACGACATCGAATACCAGATCAA |
| Pig-Mx1-R | ATGGTCCTGTCTCCTTCGG |
| Pig-OASL-F | TCCCTGGGAAGAATGTGCAG |
| Pig-OASL-R | CCCTGGCAAGAGCATAGTGT |
| Pig-ISG15-F | GACTGCATGATGGCATCGGA |
| Pig-ISG15-R | TGCACCATCAACAGGACCAT |
| Pig-PKR-F | ATTGCGAGAAGGTAGAGCGT |
| Pig-PKR-R | TTCCATTTGGATGAAAAGGCACC |
| Pig-GBP1-F | GAAGGGTGACAACCAGAACGAC |
| Pig-GBP1-R | AGGTTCCGACTTTGCCCTGATT |
| Pig-Viperin-F | GGACACTGGTACCTGTCACCTT |
| Pig-Viperin-R | TGAAGTGGTAATTGACGCTAGT |
| Pig-BST2-F | GTACACCGTTTGCAACCAGAC |
| Pig-BST2-R | CTGTTGTTCTCCTGAACGCC |
| Pig-MHC-I-F | GGCTCCAGAAATACCTGCAGAT |
| Pig-MHC-I-R | TCTCCACCAGCTCCATGTCTTG |
| Pig-MHC-II-F | ACCACAACCTCCTGGTCTGCTCT |
| Pig-MHC-II-R | CTCTCCACTCTGAGGAACCGTT |
| Pig-IRF1-F | GCAACAGATGAGGACGAG |
| Pig-IRF1-R | GCTTTCAACTTCTGGCTC |
| Pig-CXCL10- -F | TGCCCACATGTTGAGATCAT |
| Pig-CXCL10-R | CGGCCCATCCTTATCAGTAG |
| Pig-β-actin -F | GACCTGACCGACTACCTCAT |
| Pig-β-actin-R | CGTAGAGGTCCTTCCTGATGT |

Supplementary Table 2 Determination of Endotoxin Contamination

| Sample | Endotoxin  Concentration  (EU/mL) | Protein  Concentration  (μg/mL) | Endotoxin  Contamination  (EU/μg) |
| --- | --- | --- | --- |
| PoIFN-α | 0.06136 | 316.6276 | 0.00019 |
| PoIFN-γ | 0.05638 | 273.3764 | 0.00021 |

**Supplementary Table 3 ELISA for ASFV P72 antigen in the cell culture supernatants collected from IFN treated group or NC group**

| OD_450_ value | Cell supernatants of different treated groups | | | |
| --- | --- | --- | --- | --- |
|  | IFN-α | IFN-γ | IFN-α+γ | NC |
| 24 h | 0.18 | 0.26 | 0.11 | 0.96 |
| 48 h | 0.43 | 0.53 | 0.25 | 1.64 |
| 72 h | 0.66 | 0.85 | 0.32 | 2.32 |

Data is shown as the mean of three independent experiments. Data are means (n=3) ±S.E.M.

**Supplementary Table 4 IFN levels in PAM culture supernatants collected from IFN treated group or NC group**

| Time points | Antiviral activity (U/mL) in the cells | | | |
| --- | --- | --- | --- | --- |
|  | IFN-α | IFN-γ | IFN-α+γ | NC |
| 24 h | 226.74 | 70.82 | 452.82 | / |
| 48 h | 50.76 | 30.02 | 85.41 | / |
| 72 h | 20.08 | 15.19 | 45.29 | / |

Data is shown as the mean of three independent experiments. Data are means (n=3) ±S.E.M.

**Supplementary Table 5 The IFN levels in serum after the inocul**ation

| Time points | Antiviral activity (U/mL) in the cells | | |
| --- | --- | --- | --- |
|  | LDI | HDI | NC |
| 0 h | 52.05 | 416.37 | / |
| 4 h | 101.93 | 628.36 | / |
| 8 h | 25.48 | 70.28 | / |
| 12 h | 4.55 | 23.55 | / |
| 24 h | 1.41 | 3.40 | / |

Data is shown as the mean of three independent experiments. Data are means (n=3) ±S.E.M.
